# Turn the light on! Using algae to learn how chloroplasts are built

**DOI:** 10.1093/plphys/kiag694

**Published:** 2026-09-18

**Authors:** Mireia Uranga

**Affiliations:** Assistant Features Editor, Plant Physiology, American Society of Plant Biologists, Rockville, MD 20855-2768, United States; Institute for Integrative Systems Biology (I2SysBio), Universitat de València-CSIC, 46908 Paterna, Valencia, Spain

For billions of years, plants have built life from scratch using their own solar power stations. Chloroplasts are photosynthetically active plastids that use sunlight to transform inorganic matter into sugars and other organic compounds essential for living organisms. Chloroplasts originate from undifferentiated plastids that are located in meristematic cells, and their biogenesis is tightly linked to leaf development. In angiosperms, light is a key activator of the transcriptomic reprogramming needed for chloroplast formation. Arabidopsis seedlings grown in darkness accumulate nonphotosynthetic etioplasts because chlorophyll biosynthesis is blocked in the absence of light (Floris and Kühlbrandt 2021). Etioplasts accumulate high levels of protochlorophyllide (Pchlide), which associates with the light-dependent Pchlide oxidoreductase (LPOR) to form characteristic crystalline structures known as prolamellar bodies. Upon exposure to light, LPOR reduces Pchlide to chlorophyllide (Chlide) before converting it to chlorophyll (Fig. 1). A profound remodeling of the inner membrane system converts etioplasts into fully functional chloroplasts, which allows seedling de-etiolation and optimal photosynthetic capacity (Pipitone et al. 2021).

**Figure 1:**
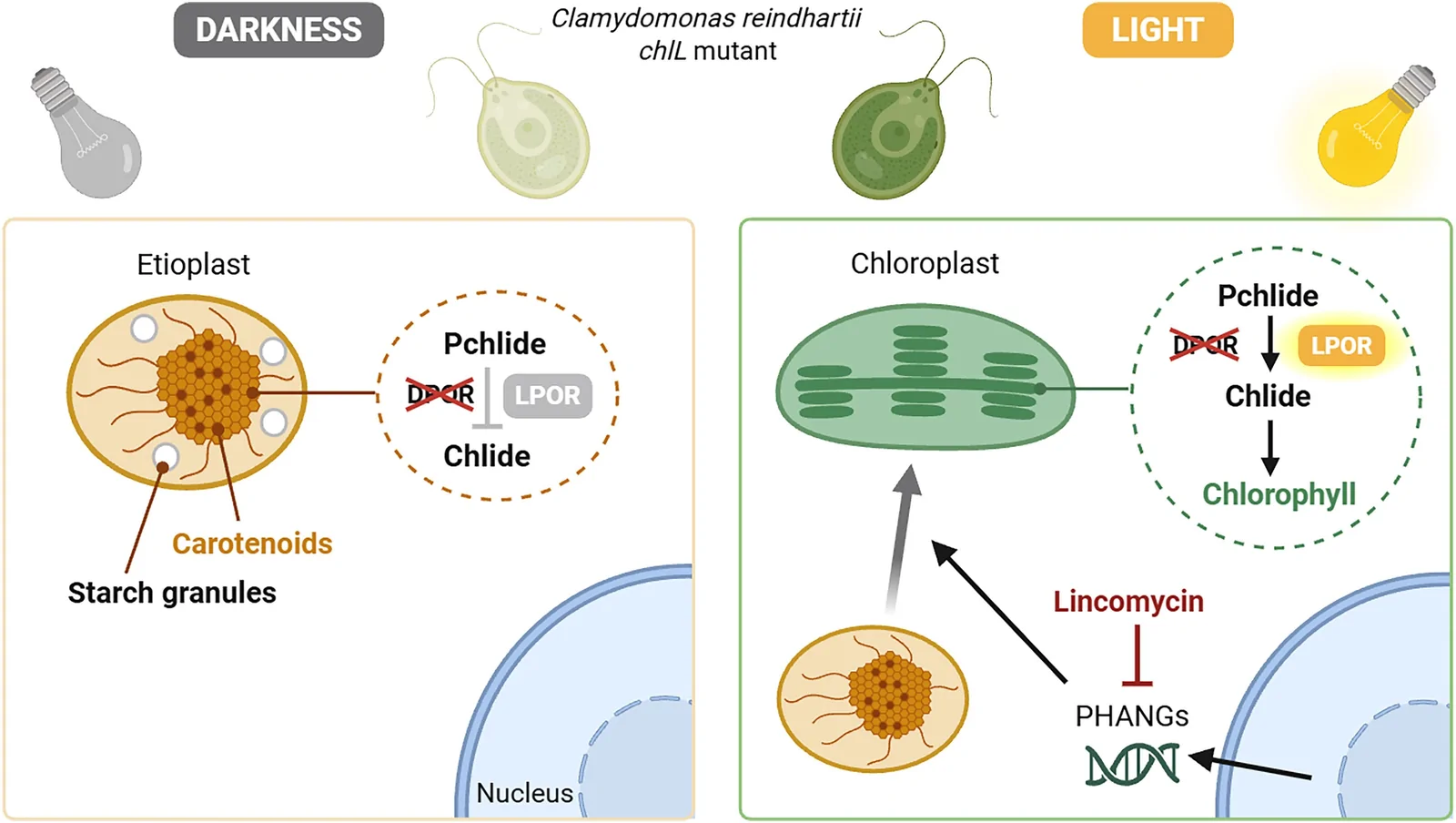
Proposed model for light-induced chloroplast biogenesis in the *Chlamydomonas reinhardtii* yellow-in-the-dark *chlL* mutant. Dark-grown *chlL* cells (left panel) exhibit etioplast-like plastids enriched in carotenoids and starch granules, because DPOR is mutated and LPOR is inactive in the absence of light. Upon illumination (right panel), active LPOR converts Pchlide to Chlide, promoting chlorophyll synthesis and chloroplast differentiation. This process is coordinated by photosynthesis-associated nuclear genes (PHANGs), whose expression is repressed by the plastid translation inhibitor lincomycin. Created in BioRender.

Unlike flowering plants, a dark-operative Pchlide oxidoreductase (DPOR) phylogenetically unrelated to LPOR allows most land plants and green algae to produce chlorophyll in the absence of light (Vedalankar and Tripathy 2019). Hence, they can develop green chloroplasts resembling those of light-grown cells even after prolonged darkness. The single chloroplast of the unicellular green alga *Chlamydomonas reinhardtii* is morphologically similar to those of higher plants, being an ideal system to study the evolution of chloroplast biogenesis (Ostermeier et al. 2024). Additionally, DPOR activity allows Chlamydomonas to grow nonphotosynthetically in acetate-containing media. Pioneering investigations into light-driven chloroplast biogenesis in Chlamydomonas used yellow-in-the-dark mutants lacking the DPOR subunit CHLL (Cahoon and Timko 2000). When grown in the dark, these mutants have undifferentiated plastids enriched in Pchlide and carotenoids, which can be converted into functional chloroplasts upon exposure to light. Unfortunately, many questions about chloroplast development in these mutants remain unanswered.

Recently in *Plant Physiology*, Ruiz-Sola et al. (2026) carried out a comprehensive characterization of light-induced chloroplast biogenesis in the yellow-in-the-dark *chlL* mutant that carries targeted mutations in the CHLL subunit (Suzuki and Bauer 1992). Initially, the authors determined the optimal growth parameters for wild-type Chlamydomonas by testing different medium compositions (minimal Tris-phosphate [TP] and TAP) and light regimes. As expected, growth in continuous light was slightly lower in TP than in TAP, while cells grown in darkness required twice as long to reach a similar developmental stage. Additionally, the authors evaluated the effect of the antibiotic lincomycin on cell growth in Chlamydomonas, commonly used in higher plants as an inhibitor of chloroplast protein synthesis. In continuous light, lincomycin notably prevented cell growth in both minimal and acetate-rich media, although no consequences were observed in darkness. This might indicate that the cell depends less on chloroplast translation during heterotrophic growth, as most of the biosynthetic functions are performed by nuclear-encoded proteins. Lincomycin treatment also reduced chlorophyll content and photosynthetic capacity regardless of the light regime and media type. Thus, these results suggest that lincomycin specifically affects chloroplast physiology, as mitochondrial respiration remained unaffected in all cases.

Ruiz-Sola et al. (2026) characterized the physiology of the *chlL* mutant by inoculating light-grown cells in liquid TAP and maintaining them under different light regimes (Fig. 1). After 5 days of continuous darkness, the mutant turned yellow due to its DPOR deficiency, which prevented chlorophyll biosynthesis in the dark. Dark-grown *chlL* cells accumulated carotenoids and starch granules and showed partially developed chloroplasts devoid of thylakoids and grana stacks. However, 48 h after light exposure and transfer to TP medium, dark-grown cells recovered a green appearance indistinguishable from that of control *chlL* grown in continuous light. The light-driven activation of LPOR rapidly converted Pchlide to Chlide, leading to chlorophyll accumulation and photosystem assembly. Additional microscopic analysis revealed the development of fully functional, cup-shaped chloroplasts featuring thylakoids piled in grana. Intriguingly, the starch granules observed in dark-grown cells disappeared upon light exposure, probably because they were used as an energy source for chloroplast biogenesis. Although the addition of lincomycin did not affect Pchlide-to-Chlide conversion, it notably impacted greening by reducing pigment accumulation, photosystem functionality, and cell division. The authors also observed that lincomycin increased baseline chlorophyll fluorescence, which might indicate the presence of free chlorophylls uncoupled from photosystems.

Because both nuclear and plastid genes encode for photosynthetic components, plastid-to-nucleus retrograde signals (RS) are essential to coordinate chloroplast biogenesis (Hwang et al. 2022). To gain insights into this process, Ruiz-Sola et al. (2026) tracked the accumulation of key photosynthetic proteins in dark-grown *chlL* cells exposed to light during a 48-h time course. Results indicated that core proteins of photosystems I and II accumulated rapidly upon illumination together with pigment synthesis. Cytochrome *b_6_f* proteins, already present in the dark, also increased in light as thylakoid membranes were formed. Conversely, the beta subunit of chloroplast ATP synthase remained constant during the time course, reflecting its essential role as an energy supplier for chloroplast formation. Protein levels in all cases were similar to those of light-grown *chlL* cells after 24 to 48 h. Due to its inhibitory role in plastid translation, lincomycin treatment dramatically impaired protein accumulation in all cases. Remarkably, it also altered the abundance of a nuclear-encoded antenna protein, suggesting that lincomycin might activate chloroplast-to-nucleus RS that prevent the expression of photosynthesis-associated nuclear genes (Fig. 1). This hypothesis was confirmed by analyzing the expression of the light-harvesting proteins LHCA6 and LHCB5, whose light-mediated induction was efficiently repressed by lincomycin.

In brief, Ruiz-Sola et al. (2026) present a comprehensive characterization of the Chlamydomonas yellow-in-the-dark *chlL* as a promising model system for studying light-driven chloroplast biogenesis in other photosynthetic lineages. Evolutionary studies using this alga will facilitate the discovery of alternative regulators and biogenic RS, previously overlooked in flowering plants. Intriguingly, the Chlamydomonas genome appears to lack master regulators of chloroplast formation previously identified in angiosperms, including GOLDEN2-LIKE proteins (Hernández-Verdeja and Lundgren 2024) and GENOMES UNCOUPLED 1 (Nishiyama et al. 2018). Gaining broader knowledge on chloroplast formation in plants will lay the groundwork for improving the photosynthetic performance of crops in the future.

## Recent related articles in Plant Physiology:

- Sun et al. (2024) found that spatial coordination of nuclear and organellar gene expression is required for photosystem assembly and chloroplast biogenesis in *Chlamydomonas*.
- Kreis et al. (2023) used TurboID-mediated proximity labeling to identify key protein networks involved in thylakoid biogenesis, photosynthetic regulation, and chloroplast stress responses in *Chlamydomonas*.

## Data Availability

None required.
